# Effect of Late Planting and Shading on Cellulose Synthesis during Cotton Fiber Secondary Wall Development

**DOI:** 10.1371/journal.pone.0105088

**Published:** 2014-08-18

**Authors:** Ji Chen, Fengjuan Lv, Jingran Liu, Yina Ma, Youhua Wang, Binglin Chen, Yali Meng, Zhiguo Zhou, Derrick M. Oosterhuis

**Affiliations:** 1 Key Laboratory of Crop Physiology & Ecology, Ministry of Agriculture, Nanjing Agricultural University, Nanjing, Jiangsu Province, PR China; 2 Department of Crop, Soil, and Environmental Sciences, University of Arkansas, Fayetteville, Arkansas, United States of America; National Key Laboratory of Crop Genetic Improvement, China

## Abstract

Cotton-rapeseed or cotton-wheat double cropping systems are popular in the Yangtze River Valley and Yellow River Valley of China. Due to the competition of temperature and light resources during the growing season of double cropping system, cotton is generally late-germinating and late-maturing and has to suffer from the coupling of declining temperature and low light especially in the late growth stage. In this study, late planting (LP) and shading were used to fit the coupling stress, and the coupling effect on fiber cellulose synthesis was investigated. Two cotton (*Gossypium hirsutum* L.) cultivars were grown in the field in 2010 and 2011 at three planting dates (25 April, 25 May and 10 June) each with three shading levels (normal light, declined 20% and 40% PAR). Mean daily minimum temperature was the primary environmental factor affected by LP. The coupling of LP and shading (decreased cellulose content by 7.8%–25.5%) produced more severe impacts on cellulose synthesis than either stress alone, and the effect of LP (decreased cellulose content by 6.7%–20.9%) was greater than shading (decreased cellulose content by 0.7%–5.6%). The coupling of LP and shading hindered the flux from sucrose to cellulose by affecting the activities of related cellulose synthesis enzymes. Fiber cellulose synthase genes expression were delayed under not only LP but shading, and the coupling of LP and shading markedly postponed and even restrained its expression. The decline of sucrose-phosphate synthase activity and its peak delay may cause cellulose synthesis being more sensitive to the coupling stress during the later stage of fiber secondary wall development (38–45 days post-anthesis). The sensitive difference of cellulose synthesis between two cultivars in response to the coupling of LP and shading may be mainly determined by the sensitiveness of invertase, sucrose-phosphate synthase and cellulose synthase.

## Introduction

Cotton fiber development is delineated into four stages: fiber initiation, elongation, secondary wall thickening and maturation [1]. Cotton fiber, which deposit almost pure cellulose into secondary cell walls, are referred to as a primary model system for cell wall biogenesis [2], [3], and many of the textile properties of cotton fiber are directly dependent on the amount and property of cellulose, which is mainly formed during secondary wall development [4]–[7].

The deposition of fiber secondary wall cellulose begin at about 16 days post anthesis (DPA) (at least 5 days prior to elongation cessation) and last around 15–35 d [6], [8], and the period would be prolonged by cool temperature [9]. Fiber cellulose synthesis is believed to be carried out by the plasma membrane-associated rosette structure [10]. In the rosette structure, sucrose synthase (SuSy) associated with the plasma membrane (M-SuSy) may form a complex with cellulose synthase (CesA) to channel carbon from sucrose into cellulose [2], [11]. In the process, sucrose is degraded by SuSy to provide uridine diphosphate glucose (UDP-glucose) for cellulose synthesis [2], [5], [12], and a portion of fructose maybe recycled to sucrose through sucrose-phosphate synthase (SPS) [5], [13]. The energy and hexoses required for the maintenance of cell growth is provided by the soluble SuSy (S-SuSy) in the cytosol [11]. Sucrose can be converted at high rates to both cellulose and callose (β-1,3-glucan) [14]. In 20 DPA cotton boll, fiber callose is codistributed with abundantly present SuSy in the fiber cell wall region (CW-SuSy) [11], [15]. The distribution of SuSy is consistent with its having a dual role in cellulose and callose synthesis in secondary-wall-stage cotton fiber [15]. In addition to SuSy, acidic invertase (either tightly bound to the cell wall or inside the vacuole) and alkaline invertase (a nonglycosylated cytosolic invertase) (INV) can also catalyze hydrolysis of sucrose [16], [17].

Cotton fiber development is restricted by declining temperature or low light in many cotton-growing areas [18]–[21]. However, these two climatic factors often appear as a combined one. Multiple cropping cotton areas (such as cotton-rapeseed or cotton-wheat double cropping systems) are popular in the Yangtze River Valley and Yellow River Valley of China [22]. Due to the competition of temperature and light resources during the growing season of double cropping system, cotton is generally late-germinating and late-maturing and has to suffer from the coupling of declining temperature and low light especially in the late growth stage, e.g., in the Yangtze River Valley, cotton often suffers from rainy and overcast weather during the early stage of flowering and boll formation, as well as from declining temperature and overcast weather during the late stage of flowering and boll formation. These sub-optimal environmental condition during fiber development may hinder cellulose synthesis in fiber [5], [23], [24], and have a negative impact on fiber quality [25]–[27].

Declining temperature hinders cellulose synthesis within cotton fiber [5]. Sucrose synthesis is a particularly cool temperature-sensitive step in the partitioning of carbon to cellulose [23], and the activities of related enzymes in cellulose synthesis (SuSy, SPS, INV) are also affected by declining temperature, and leaded to restrain cellulose synthesis and sucrose metabolism [5], [23], [28]. The activities of SuSy, SPS and INV in various plants or organs are also affected by shading, and result in the decline of biomass and yield [29]–[31], cotton grown in reduced light environments produced inferior fiber with a lower quality [32], [33].

Studies on the enzymological mechanism of carbon partitioning to cellulose synthesis have been carried out in plants under declining temperature or low light [5], [33], [34], but little is reported about the response of carbon partitioning to cellulose synthesis to the coupling of declining temperature and low light. Therefore, in the study, late planting (LP) and shading were used to fit the combined situation which cotton generally suffers from in the cotton-rapeseed or cotton-wheat double cropping systems, the impact of declining temperature and low light (formed by LP and shading) on sucrose metabolism, cellulose synthesis and related enzymes activity change during fiber secondary wall development (FSWD) were studied, and the physiological and biochemical mechanism of carbon partitioning to cellulose synthesis in response to adverse environmental conditions of declining temperature with low light would be elucidated.

## Materials and Methods

### Experimental Design

Field experiments were conducted at Pailou experimental station of Nanjing Agricultural University at Nanjing, China (32°02′N, 118°50′E), in the Yangtze River Valley in 2010 and 2011. The experimental soil was clay, mixed, thermic, Typic alfisols (udalfs; FAO luvisol) with 18.3 and 18.1 g kg^−1^ organic matter, 1.1 and 1.0 g kg^−1^ total N, 64.5 and 70.2 mg kg^−1^ available N, 17.9 and 20.3 mg kg^−1^ available P, and 102.3 and 111.1 mg kg^−1^ available K contained in 20 cm depth of the soil profile before sowing cotton in 2010 and 2011, respectively.

Two cotton (*Gossypium hirsutum* L.) cultivars, Kemian 1 which was cool temperature-tolerant and Sumian 15 which was cool temperature-sensitive [6], [35] were selected based on the categorization of cultivars widely grown in the Yangtze River Valley in its low temperature sensitivity. In the field, different environmental condition during fiber development were provided by planting cotton in different dates [6], 25 April, 25 May and 10 June in 2010 and 2011. Planting date of 25 April is comparatively appropriate to grow cotton in the Yangtze River Valley, and 25 May and 10 June are belong to late planting dates (LPD). Cotton seeds were sown in a nursery bed, and seedlings with three true leaves were transplanted to field at a spacing of 80 cm×25 cm.

When approximately 50% of flowers in the first fruiting node of the 6–7th sympodial branches of plants in each planting date bloomed, three shading treatments were imposed for the plots of each planting date, including an unshaded control (*CRLR* (crop relative light rate) 100%), mild shading (*CRLR* 80%), severe shading (*CRLR* 60%) achieved with white nylon cloth (12 m length, 7 m width, 2 m height, and two different kinds of cloth which reduced the incident light by 20% and 40%, respectively). Shading cloths were removed after cotton bolls in the first fruiting node of the twelfth synpodial branches opened. Experiments were arranged as a randomized complete block design in the field with three replications and each plot was 6 m wide and 11 m long. Furrow-irrigation was applied as needed during both seasons. Conventional insect and weed control methods were utilized as needed.

### Sampling and processing

Cotton flowers in the first or the second fruiting node of the 6–7th sympodial branches with the same anthesis date were tagged with small plastic tags listing the flowering date. About 6–8 cotton bolls in the similar size with the same anthesis date for each treatment were collected from once every 7 days starting from 10 DPA until boll opening. Cotton bolls were collected at 9:00–11:00 am, and cotton fiber were excised from bolls with a scalpel and were immediately put into liquid nitrogen for subsequent measurement.

### Weather data

Weather data were collected from the Nanjing weather station located about 6 km from the plot area. Table 1 shows the mean daily maximum temperature (MDTmax), mean daily temperature (MDT), mean daily minimum temperature (MDTmin) and mean daily radiation (MDR) during FSWD for different planting dates. FSWD was calculated from the initiation date of fiber biomass rapid-accumulation (data not shown) to the boll opening date [7], [36].

**Table 1:** Weather factors during cotton fiber secondary wall development (FSWD) under different planting dates.

Years	Planting dates	Flowering dates	Starting date of FSWD	Boll opening date	Duration of FSWD	Weather factors			
	(dd−mm)	(dd−mm)	(dd−mm)	(dd−mm)	(d)	MDT (°C)	MDTmax (°C)	MDTmin (°C)	MDR (MJ m^−2^)
2010	25-Apr	28-Jul	11-Aug	8-Sep	29	28.3	32.4	25.3	15.8
	25-May	19-Aug	8-Sep	18-Oct	41	21.4	25.5	18.5	11.5
	10-Jun	4-Sep	28-Sep	5-Nov	39	16.7	21.3	13.2	11.1
					CV(%)	26.51	21.32	31.82	20.34
2011	25-Apr	27-Jul	12-Aug	14-Sep	34	25.9	29.5	23.0	13.0
	25-May	25-Aug	14-Sep	24-Oct	41	19.9	24.5	16.6	12.5
	10-Jun	10-Sep	30-Sep	11-Nov	44	17.4	21.4	14.4	10.2
					CV(%)	20.63	16.15	24.83	12.56

MDT, MDTmax, MDTmin and MDR stand for mean daily temperature, mean daily maximum temperature, mean daily minimum temperature and mean daily radiation.

### Field microclimate measurement

Field microclimate were measured at 15, 30 and 45 DPA (15, 30 and 45 days after initiation of shading). Air temperature, relative humidity and photosynthetically active radiation (PAR) were measured every two hours from 6:00am to 6:00pm, using a Hygro-Thermometer Psychrometer (DT-8892, CEM, Shenzhen, China) to measure air temperature and relative humidity at the position of 6–7th fruiting branches. PAR was measured at the position about 0.2 m above the canopy (PAR_0_, below the shading cloth) by a Decagon AccuPAR LP-80 Ceptometer (Decagon Devices, Logan, Utah, USA). Measurements were taken only when the direct sunlight was not blocked by clouds.

### Cellulose content, sucrose content and callose content analyses

Cotton fiber was digested in an acetic-nitric reagent, and then the cellulose content was measured with anthrone according to Updegraff [37].

Sucrose was extracted and quantified by a modified method of Pettigrew [33]. About 0.3 g dry weight (DW) fiber samples were extracted with three successive 5 ml washes of 80% ethanol [5]. The ethanol samples were incubated in an 80°C water bath for 30 min.Then the samples were centrifuged at 10,000 g for 10 min, and three aliquots of supernatant were collected together for sucrose measurement [5]. The sucrose assay was conducted according to Hendrix [38].

Callose (β-1,3-glucan) was extracted and quantified by a modified method of Köhle [39]. Fiber was soaked for 2–3 h in 5 ml of ethanol to remove autofluorescent soluble material and then oven dried. The above sample (200 mg) was ground into a fine powder in liquid nitrogen followed by 5 ml of 1 N NaOH. The resulting suspension was incubated at 80°C for 30 min to solubilize the callose and centrifuged (15 min, 380 g). The supernatant was used for the callose assay. 0.6 ml of supernatant was mixed with 1.2 ml of 0.1% (w/v) aniline blue WS in water, resulting in a violet-red color. After addition of 0.63 ml of 1N HCl the color changes to deep blue, indicating neutral to acidic pH values [39]. The final pH value was adjusted by addition of 1.77 ml 1M glycine/NaOH buffer (pH 9.5) and the tubes were mixed vigorously. During the following incubation for 20 min at 50°C and further 30 min at room temperature, the aniline blue becomes almost completely decolorize [39]. Fluorescence of the assay was read in a Tecan Infinite M200 microplate reader (Tecan, Männedorf, Switzerland, excitation 400 nm, emission 510 nm, slit 5 nm). Calibration curves were established using a freshly prepared solution of the β-1,3-glucan in 1N NaOH [39].

### Enzymatic analyses

Enzyme extraction and assay were according to King [40] with minor modifications. Fiber cell samples, about 0.5 g fresh weight (FW), were ground into a fine powder in liquid nitrogen followed by grinding in cold extraction buffer (5∶1, v/w), which contained 50 mM N-(2-hydroxyethyl) piperazine -N’-(2-ethanesulfonic acid)-NaOH (Hepes-NaOH) (pH 7.5), 10 mM MgCl_2_, 1 mM ethylenediamine tetraaceticacid (EDTA), 1 mM ethyleneglycol bis-(2-aminoethylether)-tetraacetic acid (EGTA), 0.5% (w/v) bovine serum albumin (BSA), 2% (w/v) polyvinylpyrrolidone (PVP), 0.1% (v/v) Triton X-100, 2 mM dithiothreitol (DTT), and 1 mM phenylmethylsulfonyl fluoride (PMSF) as described in the previous research [5]. The resulting homogenate was centrifuged at 15,000 g for 20 min, and the supernatant was stored at 4°C for analysis [5]. All extraction procedures were carried out at 0–4°C.

SuSy activity was assayed by measuring the cleavage of sucrose [40]. Each reaction contained 20 mM piperazine-N,N’-bis (2-ethanesulfonic acid)-KOH (Pipes-KOH) (pH 6.5), l00 mM sucrose, 2 mM UDP, and 200 µl of extract in a total volume of 650 µl as described previously [5]. Reactions were started by incubating at 30°C for 30 min. The reactions were stopped with 250 µl of 0.5 M N-tric-(hydroxy-methyl) methylglycine-KOH (Tricine-KOH) (pH 8.3), which were heated for 10 min in boiling water, and the amount of fructose in SuSy reactions was determined as described before [5].

Soluble acid and alkaline invertases’ activities were measured by incubation of 100 ml of extract with 1M sucrose in 200 mM acetic acid-NaOH (pH 5.0) (acid invertase), or 100 mM sodium acetate-acetic acid (pH 7.5) (alkaline invertase), in a total volume of 2.5 ml [5]. Reactions were started by incubating at 30°C for 30 min and were stopped with 1 ml of 3,5-dinitro salicylic acid (DNS), and boiling for 5 min [5], [40]. Glucose content was measured spectrophotometrically at 540 nm.

SPS activity was assayed by measuring the synthesis of sucrose-6-P from UDP-glucose and fructose-6-P [41]. Each reaction contained 14 mM UDP-glucose, 50 mM fructose-6-P, 50 mM extraction buffers, 50 mM MgCl_2_ and 200 ml extract in a total volume of 650 ml as described in previous research [5]. Reactions were started by incubating the enzyme extracts at 30°C for 30 min and were stopped with 100 ml of 2N NaOH and 10 min of heating at 100°C to destroy unreacted hexoses and hexose phosphates [5]. After adding 1 ml of 0.1% (w/v) resorcin in 95% (v/v) ethanol, reactions were incubated for 30 min at 80°C. Sucrose-6-P content was calculated from a standard curve measured at 480 nm [5].

### Semi-quantitative RT-PCR analyses

Fiber sampling in 2011 was used in the semi-quantitative RT-PCR analyses. Total RNA was isolated from cotton fiber according to Jiang and Zhang [42]. For each reaction, 2 µg of RNA was reverse transcribed to cDNA with oligo(dT)_15_. PCR was performed in a final volume of 25 µL containing 2U of *Taq* DNA polymerase. The gene-specific primers of two cellulose synthase catalytic subunit, CesA1 and CesA2 (*GhCesA1*, U58283 and *GhCesA2*, U58284),were designed to unique regions of both cDNA (Table 2), the cotton 18srRNA gene (*Gh18srR*,U42827) [43] was used as an internal control. PCR reaction was initially denatured at 94°C for 5 min and 30 cycles at 94°C for 30 s, proper annealing temperature for 30 s and72°C for extending 30–60 s (Table 2), a final extension of RT-PCR products at 72°C for 10 min. PCR products were size-separated by electrophoresis in a 1.8% agarose gel. All photographs were statistically analyzed with the software Quantity One.

**Table 2:** Primers, T_m_, extension time and cycles in RT-PCR program.

Gene	Accession No.	Primer	T_m_ (°C)	No. of cycles	Extension time (s)	Length of amplified DNA (bp)
*GhCesA1*	U58283	Forward: 5’- TGGGTTGAATGTTAATGGT-3’	58	30	60	632
		Reverse: 5’- CAGGATACCACTTAGGGAACT-3’				
*GhCesA2*	U58284	Forward: 5'- CTGGCTTTGGTTCACTTGC-3'	58	30	60	529
		Reverse: 5'- CCGCCATTATCGTTGCTTA-3'				
*Gh18srR*	U42827	Forward: 5’-CTGAGAAACGGCTACCACAT-3’	53	25	30	500
		Reverse: 5’-CTATGAAATACGAATGCCCC-3’				

### Data analysis

OriginPro 8.0 was adopted for data processing and drawing of figures. An analysis of variance was performed using SPSS statistic package Version 17.0. The means were separated using the least significant difference (LSD) test at 5% of probability level. The coefficient of variation (CV) was calculated as the ratio of the standard deviation to the mean. Changing amplitude (Δ%) = (Treatment-Control)/Control × 100%, control is the plot of 25 April plus *CRLR* 100%. The fiber growing period delineated by the two dotted lines in figures stood for the period of fiber secondary wall rapid-thickening (FSWR), which was calculated from the initiation date to the termination date of fiber biomass rapid-accumulation. The formation of cotton fiber biomass could be described by the logistic regression model and then the initiation and termination date of the fiber biomass rapid-formation were obtained (data not shown), which represented the initiation and termination date of FSWR, respectively, and the duration was the period of FSWR [7], [36].

## Results

### Field environmental condition and field microclimate

Environmental condition of normal planting date (NPD) of 25 April was advantageous to develop cotton, and was the optimal planting date in the Yangtze River Valley [44], delaying the planting date would prolong FSWD from 29 to 41 days in 2010 and from 34 to 44 days in 2011 (Table 1). In two experimental years, MDT, MDTmax, MDTmin and MDR during FSWD decreased as planting date delayed. Fiber microclimate data in Table S1 were expressed as the mean of data measured from 6:00am to 6:00pm at 15, 30 and 45 DPA, air temperature and PAR_0_ in cotton field also decreased as planting dates delayed, but the CVs of MDT, MDTmax and MDTmin were higher than that of MDR (Table 1), it was indicated that effect of LP on temperature factors was greater than sunshine factors during FSWD. MDTmin was the primary environmental factor affected by LP, and reduced from 25.9°C to 13.2°C in 2010 and from 25.3°C to 14.4°C in 2011 (Table 1).

**Figure 1:**
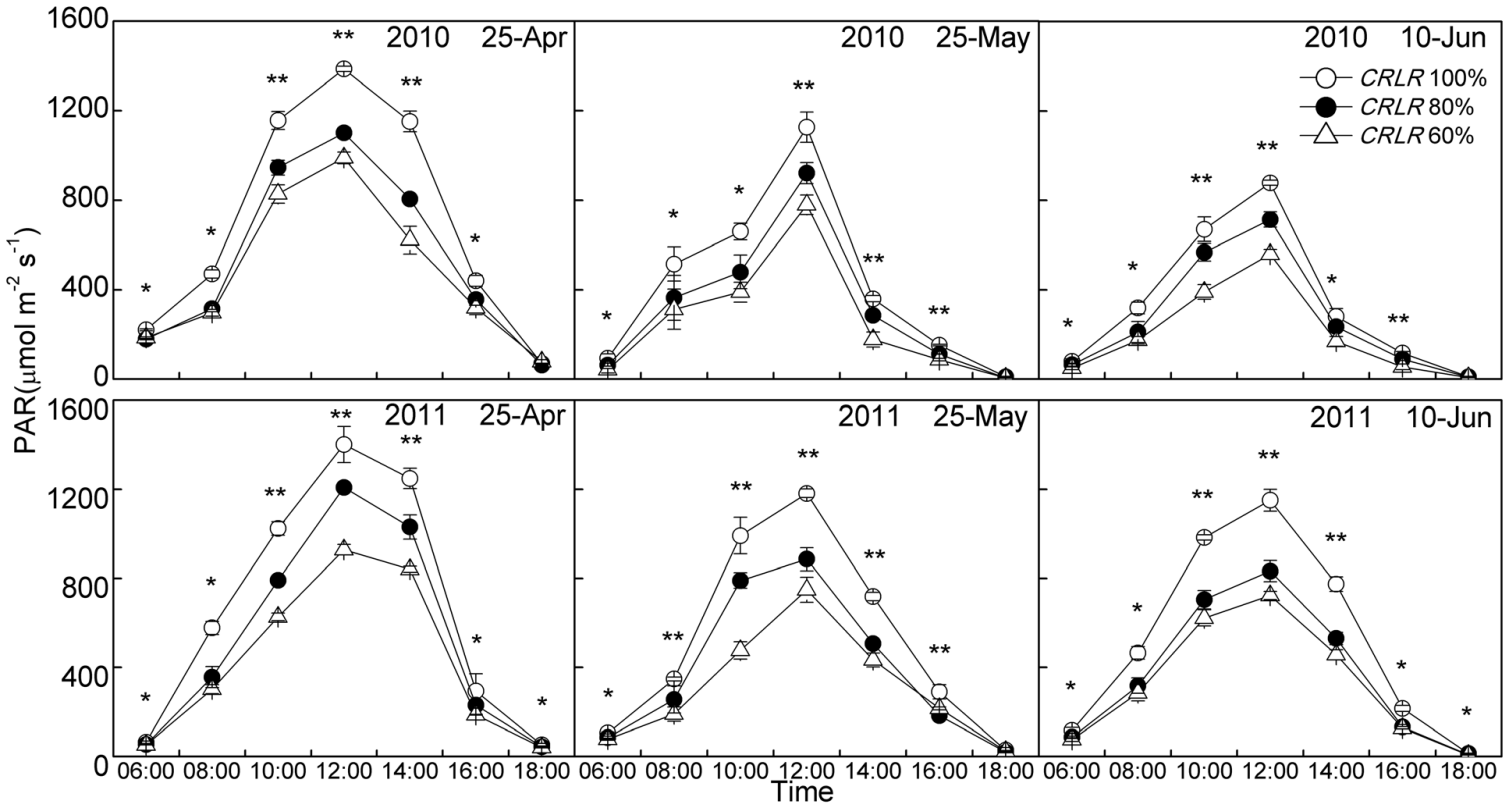
Changes of photosynthetically active radiation measured at the position about 0.2 m above the canopy (PAR_0_) at 30 DPA under the coupling of planting date and shading in 2010 and 2011. * and ** mean significant difference among three shading treatments at 0.05 and 0.01 probability levels, respectively.

**Figure 2:**
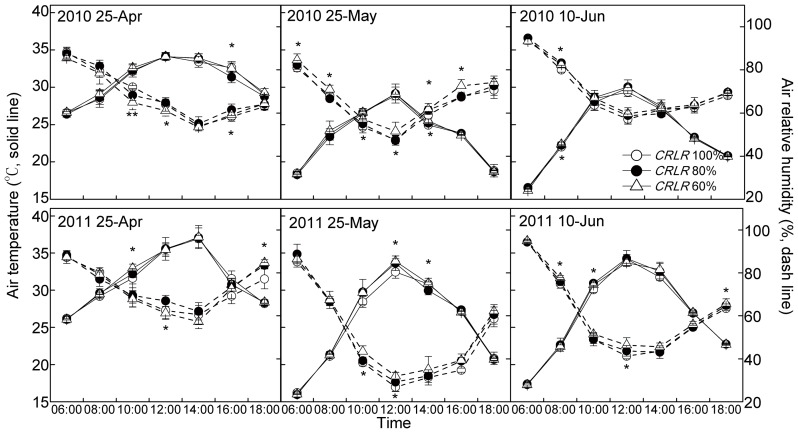
Changes of mean air temperature and mean relative humidity at 30 DPA under the coupling of planting date and shading in 2010 and 2011. * and ** mean significant difference among three shading treatments at 0.05 and 0.01 probability levels, respectively.

During 6:00am–6:00pm, PAR_0_ peaked at midday, shading significantly reduced PAR_0_ by 18%–25% for *CRLR* 80% and 35%–44% for *CRLR* 60% treatments as experimental design (Figure 1); air temperature peaked at 12:00am–2:00pm and was not significantly different between shading and normal light treatments, except one or two determination points and their numbers of each planting date with only small deviations of no more than 1.5°C (Figure 2); mean relative humidity decreased until 12:00am–2:00pm and thereafter increased (Figure 2), and shading treatments was statistically different around 10:00am–2:00pm, but the deviation of these treatments were less than 7% (Figure 2). Field microclimate data measured at 15, 30 and 45 DPA in Table S1 also showed that difference of air temperature or mean relative humidity among different shading treatments were no more than 1°C or 5%, respectively. These small differences in temperature and relative humidity would probably only be a minor effect on carbohydrate concentrations compared with the effect of 20%–40% PAR reduction, which reduced the carbohydrate levels in fiber under shading by lower photosynthetic rates because of the lower light levels as shown by Pettigrew [33]. Therefore, the decline of PAR_0_ was the key reason for the adverse effect on cotton fiber development caused by shading.

### Sucrose, cellulose and callose contents in cotton fiber

**Figure 3:**
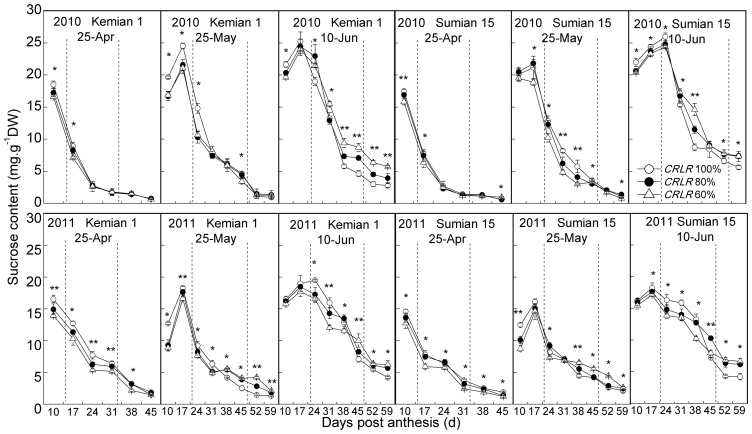
Changes of sucrose contents in cotton fiber of two cultivars under the coupling of planting date and shading in 2010 and 2011. The fiber growing period delineated by the two dotted lines stand for the period of fiber secondary wall rapid-thickening (FSWR). * and ** mean significant difference among three shading treatments at 0.05 and 0.01 probability levels, respectively.

During fiber development, sucrose contents in cotton fiber declined from 10 DPA under NPD_25-Apr_, but there were a peak value which occurred at 17 and 24 DPA under LPD_25-May_ and LPD_10-Jun_, respectively (Figure 3). LP enhanced sucrose content in cotton fiber compared to the normal planting. Compared to *CRLR* 100%, under NPD_25-Apr_, sucrose contents of *CRLR* 80% and *CRLR* 60% decreased. Sucrose in cotton fiber after 38 DPA under NPD_25-Apr_ had already been depleted, but there was surplus sucrose in the developing fiber of 59 DPA in LPD_10-Jun_ (Figure 3). Under LPD, fiber sucrose contents of *CRLR* 80% and *CRLR* 60% decreased before 31 DPA, but after 31 DPA the sucrose contents of *CRLR* 100% were lower than that of *CRLR* 80% and *CRLR* 60% (Figure 3). The maximum sucrose content in cotton fiber can reflect the amount of available sucrose in developing fiber, and the minimum sucrose content show the residual sucrose content in mature fiber [5]. Shading could decrease the maximum sucrose content by 3.5%–15.7%, while the maximum sucrose and minimum sucrose content under LPD increased 10.4%–48.5% and 125.8%–1349.0%, respectively, compared to normal planted cotton (Table 3, Table 4). The maximum sucrose content under the coupling of LP and shading did not increase as much as that under the same planting date without shading. In contrast, the minimum sucrose content under LPD_10-Jun_ plus *CRLR* 60% reached the highest (Table 3). The CVs of the maximum and minimum sucrose contents in two cultivars caused by LP were higher than that caused by shading, it was indicated that the effect of LP on fiber sucrose content was greater than that of shading, and the CVs caused by the coupling of LP and shading were similar to LP. Compared to the maximum sucrose content, the minimum sucrose content was more susceptible to LP or shading, and the trend was consistent between two cultivars (Table 3). The CVs and its changing amplitude (Δ%) of the maximum sucrose content response to the coupling of LP and shading in Sumian 15 was higher than that of Kemian 1, but the CVs and its Δs of the minimum sucrose content between two cultivars was different in two years (Table 3, Table 4).

**Table 3:** Maximum/minimum sucrose, maximum callose and final cellulose contents in cotton fiber and their analysis of variance under the coupling of planting date and shading in 2010 and 2011.

Planting dates	*CRLR*	Maximum sucrose content		Minimum sucrose content		Maximum callose content		Final cellulose content	
(dd–mm)	(%)	(mg g^−1^DW)		(mg g^−1^DW)		(mg g^−1^DW)		(%)	
		2010	2011	2010	2011	2010	2011	2010	2011
**Kemian 1**									
25-Apr	100	18.5 a	16.5 a	0.7 a	1.5 a	7.7 b	5.6 c	92.2 a	91.6 a
	80	17.3 b	14.9 b	0.7 a	1.9 a	7.6 b	9.6 b	91.2 a	89.0 a
	60	16.6 c	13.9 c	0.6 a	1.4 a	11.4 a	11.5 a	87.0 b	90.6 a
25-May	100	24.5 a	18.3 a	4.6 a	3.9 a	8.3 b	7.0 c	85.3 a	82.3 a
	80	21.5 b	17.6 b	4.4 a	4.1 a	8.1 b	9.7 b	85.0 a	77.0 b
	60	21.1 b	16.6 c	3.4 a	2.5 b	12.2 a	12.6 a	83.1 b	76.8 b
10-Jun	100	25.2 a	19.5 a	4.6 c	7.1 c	9.4 b	7.0 c	78.4 a	76.6 a
	80	24.4 b	19.0 a	7.0 b	8.2 b	9.3 b	11.6 b	76.8 b	73.3 b
	60	23.9 c	17.7 b	8.7 a	10.0 a	12.8 a	13.3 a	75.4 c	73.1 b
	CV_P_(%)	16.23	8.25	68.92	67.66	10.39	13.09	8.09	9.07
	CV_S_(%)	5.60	8.64	6.97	14.50	24.11	34.11	3.90	1.45
	CV_P×S_(%)	15.53	10.66	73.90	70.52	20.86	27.90	7.09	9.19
>**Sumian 15**									
25-Apr	100	17.5 a	14.6 a	0.6 a	1.9 a	8.4 b	8.1 b	86.2 a	88.0 a
	80	16.9 b	13.6 b	0.6 a	1.3 a	12.7 a	8.2 b	85.6 ab	87.4 a
	60	15.8 c	12.8 c	0.9 a	1.2 a	13.1 a	13.0 a	84.7 b	86.0 a
25-May	100	21.2 a	16.1 a	3.6 a	4.3 b	9.3 c	9.4 b	80.4 a	81.1 a
	80	21.8 a	15.0 b	3.0 a	4.2 b	14.2 b	9.2 b	77.2 ab	79.1 ab
	60	19.5 b	14.6 b	3.3 a	5.6 a	15.1 a	13.8 a	76.5 b	77.1 b
10-Jun	100	26.0 a	18.2 a	8.4 a	7.2 b	10.7 c	10.3 c	72.0 a	69.6 a
	80	24.8 b	17.6 ab	9.1 a	10.3 a	14.7 b	11.1 b	70.0 ab	66.5 ab
	60	24.4 b	17.1 b	9.0 a	8.0 b	16.2 a	14.4 a	68.0 b	65.6 b
	CV_P_(%)	19.74	11.31	94.45	59.86	12.20	12.13	8.98	11.68
	CV_S_(%)	5.05	6.57	30.95	24.40	22.86	28.57	0.88	1.18
	CV_P×S_(%)	17.73	12.07	84.28	65.09	21.34	21.96	8.83	11.29

DW, dry weight; Values followed by the different letters within a column are significantly different at 0.05 probability level; CV_S_ was calculated from the data of three shading treatments in normal planting date of 25 April; CV_P_ was calculated from the data of three planting dates and CV_P×S_ was calculated from the data of all treatments; * and ** mean significant difference at 0.05 and 0.01 probability levels, respectively; NS means non-significant differences.

**Table 4:** The changing amplitude (Δ%) of maximum/minimum sucrose, maximum callose and final cellulose contents in cotton fiber under the coupling of planting date and shading in 2010 and 2011.

Treatments	Maximum sucrose content		Minimum sucrose content		Maximum callose content		Final cellulose content	
	2010	2011	2010	2011	2010	2011	2010	2011
**Kemian 1**								
*CRLR* 80%	−6.8	−9.6	9.2	25.6	−1.3	73.0	−1.1	−2.8
*CRLR* 60%	−10.4	−15.7	−4.7	−2.6	47.6	106.7	−5.6	−1.1
25-May	32.4	10.7	584.6	161.0	7.2	26.6	−7.5	−10.2
25-May+*CRLR* 80%	16.3	6.8	554.0	174.4	4.5	75.0	−7.8	−15.9
25-May+*CRLR* 60%	13.8	0.5	405.7	68.2	58.4	127.8	−9.9	−16.2
10-Jun	36.4	18.0	584.9	376.5	22.4	26.8	−15.0	−16.4
10-Jun+*CRLR* 80%	32.0	14.8	944.7	455.2	20.4	108.4	−16.7	−20.0
10-Jun+*CRLR* 60%	29.2	7.3	1191.3	573.4	66.8	138.9	−18.2	−20.2
**Sumian 15**								
*CRLR* 80%	−3.5	−6.6	−0.9	−28.9	51.7	1.8	−0.7	−0.7
*CRLR* 60%	−9.5	−12.3	64.5	−36.1	55.6	60.6	−1.7	−2.3
25-May	21.1	10.4	513.6	125.8	10.8	16.6	−6.7	−7.8
25-May+*CRLR* 80%	24.8	3.3	422.1	121.8	69.8	13.5	−10.4	−10.1
25-May+*CRLR* 60%	11.6	0.2	466.5	195.3	80.5	70.2	−11.3	−12.4
10-Jun	48.5	25.2	1349.0	281.9	27.3	27.7	−16.5	−20.9
10-Jun+*CRLR* 80%	41.9	21.2	1468.0	446.5	75.5	37.0	−18.8	−24.4
10-Jun+*CRLR* 60%	39.5	17.7	1445.5	322.1	92.9	77.9	−21.1	−25.5

Δ% = (Treatment-Control)/Control × 100%, control is the plot of 25 April + *CRLR* 100%.

**Figure 4:**
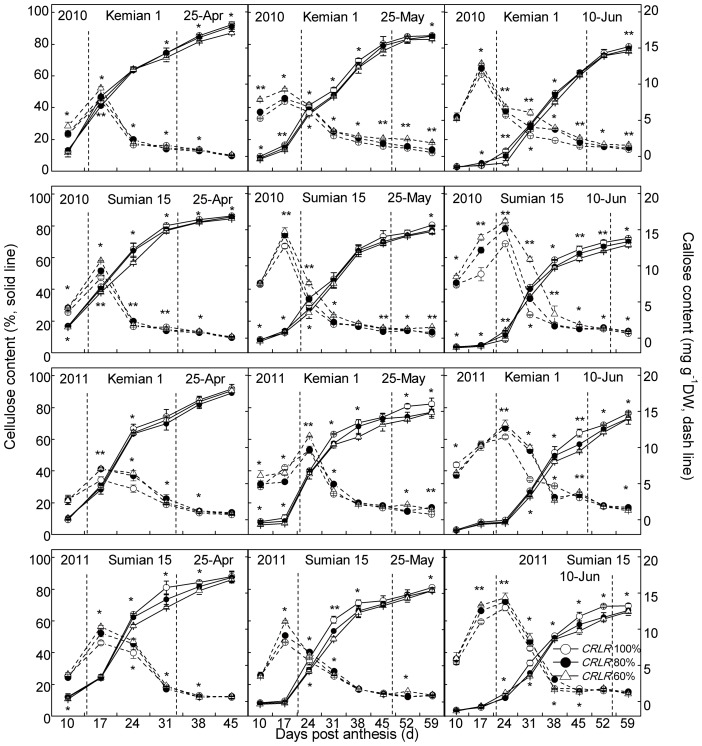
Changes of cellulose and callose contents in cotton fiber of two cultivars under the coupling of planting date and shading in 2010 and 2011. The fiber growing period delineated by the two dotted lines stand for the period of fiber secondary wall rapid-thickening (FSWR). * and ** mean significant difference among three shading treatments at 0.05 and 0.01 probability levels, respectively.

Carbon from sucrose can be converted at high rate to both cellulose and callose (β-1,3-glucan) [14]. In this study, β-1,3-glucan content in cotton fiber was low at 10 DPA and rose abruptly at approximately the time as the onset of secondary wall cellulose synthesis as also reported by Maltby et al. [45]. Callose content increased from 10 DPA to 17–24 DPA when the peak appeared (Figure 4), and then declined significantly. LP and shading could increased fiber callose content, which reached the maximum under the coupling of LP and shading (Figure 4, Table 3). The period of FSWR was 17–31 DPA under NPD_25-Apr_, and were 24–45 DPA and 24–52 DPA under LPD_25-May_ and LPD_10-Jun_, respectively. Meanwhile, the fiber cellulose rapid-accumulation was almost the same (Figure 4). Cellulose content in cotton fiber increased from 10 DPA, and compared to NPD_25-Apr_ which the initiation of fiber cellulose fast-accumulation began at 17 DPA, LPD_10-Jun_ delayed to 24 DPA and restrained cellulose synthesis. Shading under all planting dates decreased fiber cellulose contents (Figure 4), but the decreasing extents were different in different fiber development stages and planting dates. Shading under NPD_25-Apr_ had greater impacts on cellulose synthesis during 24–31 DPA, while shading under LPD were during 38–45 DPA. At the mature stage (59 DPA), *CRLR* 80% and *CRLR* 60% decreased cellulose content by 0.7%–2.8% and 1.1%–5.6% under NPD_25-Apr_, respectively, while LPD_25-May_ and LPD_10-Jun_ decreased by 6.7%–10.2% and 15.0%–20.9%, respectively (Table 4). The final cellulose content under the coupling of LPD_10-Jun_ and *CRLR* 60% was the lowest which decreased 18.2%–25.5% (Table 3). The CVs of the maximum callose contents of two cultivars caused by shading were higher than that caused by LP and the coupling of LP and shading, it was indicated that the effects of shading on maximum callose contents were greater than LP and the coupling of LP and shading. In contrast, the effects of LP on final cellulose contents were greater than shading, but close to the coupling of LP and shading (Table 3). The CVs and Δs of the final cellulose content response to the coupling of LP and shading in Sumian 15 were higher than Kemian 1 (Table 3, Table 4), it was indicated that cellulose content of Sumian 15 was more sensitive to the coupling of LP and shading than Kemian 1.

### Activity changes of related cellulose synthesis enzymes during fiber secondary wall development

Fiber SuSy activity decreased during fiber development (Figure S1). During FSWR, SuSy activity was the lowest under LPD_25-May_ and rose again under LPD_10-Jun_ (Table 5). Shading decreased the SuSy activities under all planting dates. In the period of FSWR, the decline caused by shading under NPD_25-Apr_ and LPD_10-Jun_ were larger than LPD_25-May_ (Figure S1) and the effect of shading under LPD_10-Jun_ on the Δs of SuSy activities were opposite in two years (Table 6).

**Table 5:** Average activities of sucrose phosphate synthase (SPS), sucrose synthase (SuSy), acidic invertase (Acidic INV) and alkaline invertase (Alkaline INV) in cotton fiber during fiber secondary wall rapid-thickening (FSWR) and their analysis of variance under the coupling of planting date and shading in 2010 and 2011.

Planting dates	*CRLR*	SPS		SuSy		Acidic INV		Alkaline INV	
(dd-mm)	(%)	(mg sucrose g^−1^FW h^−1^)		(mg fructose g^−1^FW h^−1^)		(mg glucose g^−1^FW h^−1^)		(mg glucose g^−1^FW h^−1^)	
		2010	2011	2010	2011	2010	2011	2010	2011
**Kemian 1**									
25-Apr	100	13.9 a	10.0 a	71.2 a	72.8 a	63.7a	78.1 a	54.8 a	70.8 a
	80	14.2 a	8.6 b	68.4 b	63.8 b	62.2 b	74.9 b	52.6 ab	65.1 b
	60	12.7 b	7.8 c	64.3 c	63.1 b	57.8 c	71.2 c	50.8 b	64.8 b
25-May	100	13.7 a	9.8 a	51.7 a	50.4 a	70.9 a	73.7 a	55.9 a	69.5 a
	80	13.6 a	9.2 b	51.8 a	45.1 b	66.1 b	68.4 b	52.7 b	66.2 b
	60	12.9 b	8.4 c	48.0 b	43.6 c	61.6 c	64.1 c	52.0 c	63.3 c
10-Jun	100	11.2 a	7.8 a	94.6 a	66.7 a	118.3 a	104.4 a	73.8 a	88.6 a
	80	10.5 a	6.8 b	88.7 b	59.8 b	110.0 b	101.3 b	69.7 b	85.5 b
	60	9.8 b	6.3 c	88.8 b	59.7 b	103.2 c	95.6 c	68.7 b	85.6 b
	CV_P_(%)	11.68	13.30	29.61	18.35	35.20	19.46	17.37	14.02
	CV_S_(%)	6.04	12.50	5.11	8.14	4.95	4.65	3.77	5.06
	CV_P×S_(%)	12.86	15.38	25.33	17.06	30.21	18.51	15.30	14.04
**Sumian 15**									
25-Apr	100	14.4 a	9.6 a	66.3 a	73.1 a	73.2 a	77.0 a	50.3 a	70.7 a
	80	12.7 b	9.3 a	62.6 b	69.5 b	71.0 b	71.3 b	48.4 b	68.3 b
	60	11.0 c	8.3 b	59.5 c	66.0 c	67.6 c	66.7 c	47.3 b	68.1 b
25-May	100	12.8 a	9.6 a	50.1 a	49.6 a	73.4 a	81.4 a	61.5 a	65.3 a
	80	12.5 ab	9.1 b	47.2 b	47.9 b	73.9 a	77.0 b	59.8 b	62.4 b
	60	11.9 b	9.1 b	47.0 b	43.9 c	67.6 b	70.4 c	57.0 c	62.4 b
10-Jun	100	9.0 a	6.6 a	88.0 a	68.6 a	105.6 c	130.2 a	71.0 c	77.5 c
	80	8.8 ab	5.9 b	84.7 a	63.8 b	107.3 b	124.2 b	72.8 b	79.8 b
	60	8.2 b	5.8 b	74.8 b	59.4 c	108.5 a	123.9 b	75.3 a	84.4 a
	CV_P_(%)	23.24	19.66	27.95	19.54	22.16	30.68	17.01	8.59
	CV_S_(%)	13.53	7.15	5.38	5.08	4.00	7.23	3.12	2.09
	CV_P×S_(%)	19.12	19.36	23.98	17.61	21.85	28.98	17.76	11.08

FW, fresh weight; Values in the same planting date followed by the different letters within a column are significantly different at 0.05 probability level; CV_S_ was calculated from the data of three shading treatments in normal planting date of 25 April, CV_P_ was calculated from the data of three planting dates and CV_P×S_ was calculated from the data of all treatments; * and ** mean significant difference at 0.05 and 0.01 probability levels, respectively; NS means non-significant differences.

**Table 6:** The changing amplitude (Δ%) of average activities of sucrose phosphate synthase (SPS), sucrose synthase (SuSy), acidic invertase (Acidic INV) and alkaline invertase (Alkaline INV) in cotton fiber during fiber secondary wall rapid-thickening (FSWR) under the coupling of planting date and shading in 2010 and 2011.

Treatments	SPS		SuSy		Acidic INV		Alkaline INV	
	2010	2011	2010	2011	2010	2011	2010	2011
**Kemian 1**								
*CRLR* 80%	1.8	−13.5	−3.9	−12.4	−2.4	−4.1	−3.9	−8.1
*CRLR* 60%	−9.2	−21.8	−9.7	−13.4	−9.2	−8.9	−7.2	−8.5
25-May	−1.7	−1.5	−27.3	−30.8	11.4	−5.7	2.0	−1.9
25-May+*CRLR* 80%	−2.3	−8.4	−27.3	−38.1	3.8	−12.4	−3.8	−6.6
25-May+*CRLR* 60%	−7.5	−15.7	−32.6	−40.1	−3.2	−18.0	−5.1	−10.7
10-Jun	−19.6	−21.9	32.9	−8.4	85.8	33.7	34.7	25.1
10-Jun+*CRLR* 80%	−24.3	−32.1	24.6	−17.9	72.7	29.6	27.2	20.7
10-Jun+*CRLR* 60%	−29.9	−37.4	24.7	−18.1	62.0	22.4	25.5	20.9
**Sumian 15**								
*CRLR* 80%	−11.9	−2.4	−5.6	−4.9	−3.0	−7.4	−3.8	−3.4
*CRLR* 60%	−23.8	−12.8	−10.2	−9.7	−7.7	−13.4	−6.0	−3.7
25-May	−10.8	0.3	−24.5	−32.1	0.3	5.7	22.2	−7.7
25-May+*CRLR* 80%	−13.4	−4.9	−28.7	−34.5	1.0	0.0	18.9	−11.8
25-May+*CRLR* 60%	−17.5	−5.1	−29.2	−39.9	−7.7	−8.6	13.3	−11.7
10-Jun	−37.8	−30.5	32.8	−6.1	44.3	69.0	41.2	9.5
10-Jun+*CRLR* 80%	−38.8	−37.8	27.8	−12.7	46.6	61.3	44.6	12.9
10-Jun+*CRLR* 60%	−43.0	−39.5	12.9	−18.7	48.2	60.9	49.8	19.4

Δ% = (Treatment-Control)/Control × 100%, control is the plot of 25 April + *CRLR* 100%.

Acidic INV in cotton fiber was higher than alkaline invertase, from the point of view of CVs, acidic INV was more susceptible to LP or shading, and both of their activities decreased during fiber development (Figure S2–S3, Table 5). Under LPD, the fiber acidic and alkaline INV activities increased and remained at a high rate at the end of fiber development. During FSWR, shading decreased acidic and alkaline INV activities of Kemian 1, shading also decreased acidic and alkaline INV activities of Sumian 15 under planting date of 25 April and 25 May, but increased the activities under LPD_10-Jun_ (Figure S2–S3).

Fiber SPS activity increased and peaked at 24 or 31 DPA, and then decreased during fiber development. LP delayed the peaks to 38, 45 or 52 DPA and decreased the peak values (Figure S4). During FSWR, SPS activity decreased under shading and the peak was delayed under LPD_25-May_ in 2010. SPS activity under NPD_25-Apr_ was similar to LPD_25-May_ during FSWR, however decreased notably under LPD_10-Jun_ (decreasing 19.6%–37.8%). Under the coupling of shading and LPD_10-Jun_, SPS activities decreased by 24.3%–43.0%, which were more than shading or LP alone, and the decreasing amplitude of Sumian 15 (decreasing 37.8%–43.0%) was larger than that of Kemian 1 (decreasing 24.3%–37.4%) (Figure S4, Table 5, Table 6).

After analysing the CVs of mean sucrose metabolism enzyme activities (such as SuSy, SPS and acidic/alkaline INV), it was found that the effects of LP on sucrose metabolism enzymes were greater than shading during FSWR, and were similar to the effects of the coupling of LP and shading. Among the four kinds of sucrose metabolism enzymes, SPS was the most significant affected by shading, and acidic INV was the most significant affected by LP and by the coupling of LP and shading (Table 5).

### Gene expression of cellulose synthase in cotton fiber

**Figure 5:**
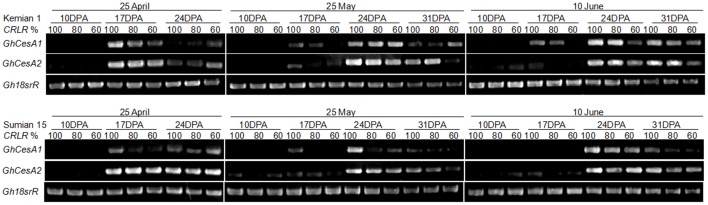
Gene expressions of two cellulose synthase catalytic subunits (CesA1 and CesA2) in cotton fiber of two cultivars under the coupling of planting date and shading in 2011.

**Figure 6:**
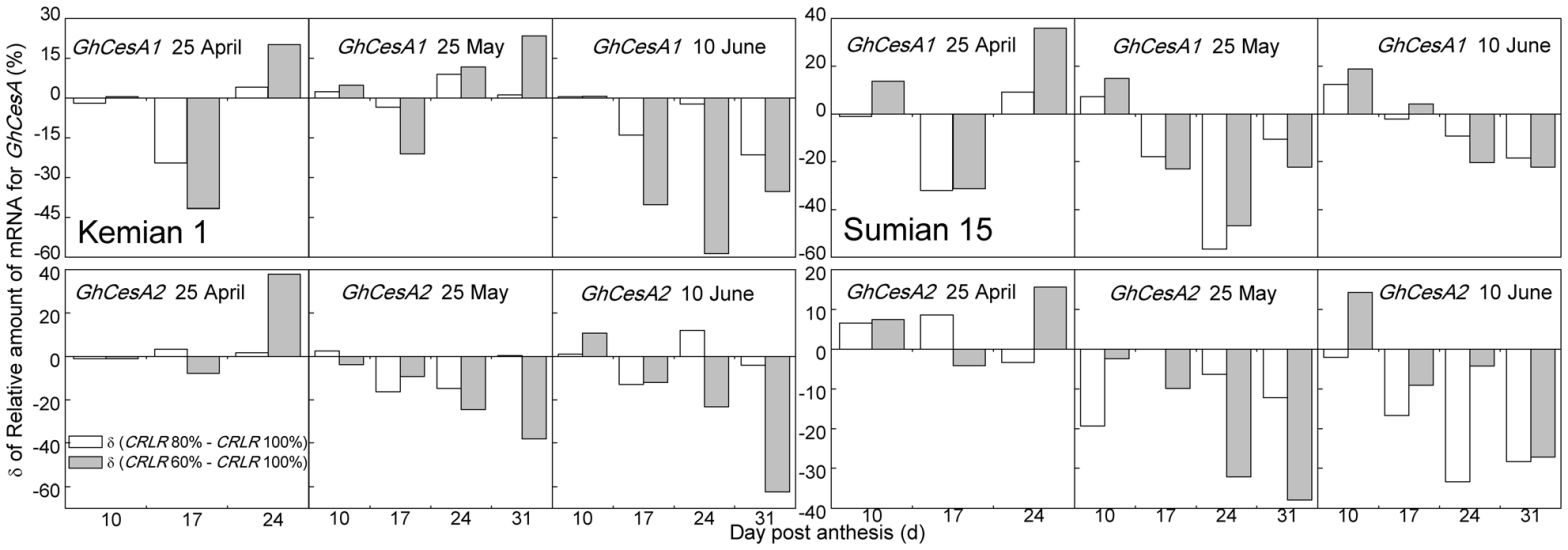
δ of relative amount of mRNA for two cellulose synthase catalytic subunits (CesA1 and CesA2) in cotton fiber of two cultivars under the coupling of planting date and shading in 2011. δ = (*CRLR* 80% - *CRLR* 100%) or (*CRLR* 60% - *CRLR* 100%).

The peak of *GhCesA1* and *GhCesA2* expression under NPD_25-Apr_ appeared around 17 DPA, and delayed to 24 DPA under LPD (Figure 5). Under NPD_25-Apr_ and LPD_25-May_, shading delayed the *GhCesA1* expression in Kemian 1, and then shading restrained the expression in LPD_10-Jun_ (Figure 5–6). Compared to Kemian 1, shading delayed the *GhCesA1* expression of Sumian 15 under NPD_25-Apr_ and shading restrained the expression under LPD.

Gene expression intensity and duration of *GhCesA1* were lower than that of *GhCesA2* in two cultivars, it was indicated that the coupling of LP and shading might have a more adverse effect on *GhCesA1* than *GhCesA2*. Under NPD_25-Apr_, shading delayed the *GhCesA2* expression, and appeared to restrain the *GhCesA2* expression under LPD. The postponing or restraining extent under *CRLR* 60% was more serious than under *CRLR* 100% and *CRLR* 80% (Figure 5–6).

## Discussion

Cotton fiber development is affected by cool temperature [5], [36], [46] and low light [19], [20], [33], [47], which often occur together [7], [48]. In order to fit the coupling situation of declining temperature and low light which cotton generally suffers from in the cotton-rapeseed or cotton-wheat double cropping systems, the couping of LP and shading experiments were designed and carried out in 2010 and 2011. The results showed that MDTmin was the primary environmental factor affected by LP during FSWD (Table 1), and shading under three planting dates significantly reduced PAR_0_ by 18%–25% and 35%–44% under *CRLR* 80% and *CRLR* 60%, respectively (Figure 1). LPD_25-May_ and LPD_10-Jun_ (MDTmin_FSWD_ of 18.5 and 13.2°C in 2010, 16.6 and 14.4°C in 2011) plus *CRLR* 80% and *CRLR* 60% resulted in an extending of cotton fiber development period and affected cellulose synthesis (Tables 1 and 3).

Toward the end of fiber elongation, secondary wall deposition begins via enhanced cellulose synthesis, while fiber callose synthesis reaches the peak in this period [2], [14], [45]. Sucrose is the substrate that is required for high-rate callose and secondary-wall cellulose synthesis, and sucrose metabolism is sensitive to cool temperature or shading [2], [14], [41], [33]. In this study, the coupling of LP and shading had more adverse impacts on cellulose synthesis during 38–45 DPA than before 31 DPA (Figure 4). Under LPD, shading decreased sucrose content before 31 DPA, and then increased it after 31 DPA in fiber (Figure 3, Table 3). The initial decline of sucrose content under shading was probably caused by lower photosynthetic rates [33], and the next increasing of sucrose content under LPD was a self-regulating phenomenon and the reduced sucrose transformation rate [5]. In developing fiber during 38–45 DPA under the coupling of LP and shading, the greater increasing range of residual sucrose content and decreasing range of cellulose content indicated that compared to single stress, the sucrose transforming ability declined more under combined stress, and more obviously during 38–45 DPA. On the other hand, sucrose could not be effectively used for cellulose synthesis under cool temperature [5] and callose synthesis can replace cellulose synthesis after wounding [2]. Under NPD_25-Apr_, the transformation from sucrose to cellulose and callose advanced simultaneously, and fiber cellulose started to accumulate rapidly before 17 DPA (cellulose contents were about 40%, Figure 4), but sucrose converted to callose more than to cellulose under shading. In contrast, under LPD, although fiber sucrose content was high during 10–17 DPA, the conversion rate of sucrose to callose was much greater than to cellulose, with lower cellulose contents about 10% before 24 DPA, shading under LPD exacerbated the situation (Figure 4). The result indicated that in the early stage of FSWD (before 24 DPA), compared to LP or shading, there was more abundant sucrose in cotton fiber under the coupling of LP and shading, however, carbon from sucrose was converted mainly to callose instead of cellulose. Whereas in the later stage of FSWD (after 38 DPA), carbon in fiber stagnated in the form of sucrose and the partitioning to cellulose synthesis decreased, and the coupling of LP and shading made it more serious.

The postponing or restraining trend of *GhCesA* expression under the coupling of LP and shading were corresponding with the downward trend of cellulose (Figure 4 and Table 4). The cellulose synthase complex was sensitive to adverse environmental effects and which directly affected cellulose synthesis. As to cool temperature-sensitive cultivar Sumian 15, shading appeared to restrain *GhCesA* expression under LPD_25-May_, earlier than shading under LPD_10-Jun_ restraining the expression in relative cool temperature-tolerant cultivar Kemian 1, it was indicated that the *GhCesA* in Sumian 15 was more sensitive to environmental change, and the postponing or restraining extent was more serious as increasing shading degree (Figure 5).

In cotton fiber, there were many sucrose metabolism enzymes contributing to cellulose synthesis besides CesA [2], [12], [49]. Sucrose is degraded to provide UDP-glucose for cellulose synthesis by SuSy, which is the critical partner in high-rate secondary-wall cellulose synthesis [2], [12], [49]. Only M-SuSy or CW-SuSy protein associated with CesA in the plasma membrane-associated rosette structure possess β-1,4-glucan (cellulose) synthesis activity [10], [11]. Compared to NPD_25-Apr_, the restrained activity of SuSy during FSWR under LPD_25-May_ was caused by shading and declining temperature, and the increasing activity under the coupling of shading and LPD_10-Jun_ (MDTmin was the lowest in this experiment) might be due to a large part of M-SuSy becoming S-SuSy. The enhanced S-SuSy degraded sucrose for maintenance and survival metabolism through glycolysis instead of contributing to cellulose synthesis [2], [5], [11].

Consistent with the results of Shu et al. [5], activities of another sucrose degrading enzymes acidic/alkaline INV in cotton fiber of two cultivars increased under LPD, and was reduced by shading under the planting date of 25 April and 25 May, but shading under LPD_10-Jun_ (MDTmin_FSWD_ reached the lowest of 16.6 and 14.4°C in 2010 and 2011) increased INV activities of Sumian 15. In contrast, shading under LPD_10-Jun_ reduced INV activities of Kemian 1 (Table 5), the probable reason was that Sumian 15 was a cool temperature-sensitive cultivar, while acidic INV was the most affected enzyme by the coupling of LP and shading (Table 5), INV in fiber was more sensitive to the coupling of LP and shading than Kemian 1, the increasing extent of INV activity in Sumian 15 was greater as the coupling stress became heavier (Table 6). However, abundant fructose produced by the increased INV activity under the coupling of LP and shading would inhibit the ability of M-SuSy [2], [5] and have an adverse effect on cellulose synthesis.

As the enzyme synthesizing sucrose in cotton fiber, SPS is very sensitive to cool temperature and the activity is hindered under adverse environmental conditions [2], [5]. SPS was also the most sensitive to shading among four sucrose metabolism enzymes (SuSy, SPS, acidic/alkaline INV, Table 5). Flux from fructose to sucrose might be hindered due to the decline of SPS activity, leading to fructose increasing in fiber, which further suppressed M-SuSy activity, and resulting in an adverse effect on cellulose synthesis. Under the coupling of LP and shading, SPS activity in fiber decreased, and the activity peak was delayed to 38, 45 or 52 DPA, similar to the time when the coupling of LP and shading had greater effects on cellulose synthesis (Figure 4 and Figure S4), it was indicated that the decline of SPS activity and its peak delay may be the reason why cellulose synthesis was sensitive to the combined stress during the later stage of FSWD (38–45 DPA).

The basic mechanisms regulating cellulose synthesis in different cotton cultivars are believed to be similar [6], but cotton cultivars have different levels of sensitivity in response to adverse environmental stress [4], [33]. It has been shown that Sumian 15 which was cool temperature-sensitive, and Kemian 1 was partially tolerant to cool temperature [6], [35]. In our research, Sumian 15 had a higher negative response of fiber cellulose to the coupling of LP and shading compared to Kemian 1 (Table 4). The decreasing range of SPS activity in Sumian 15 was greater, and CesA was more sensitive to the coupling of LP and shading than Kemian 1 (Figure 5, Table 6). In contrast, Kemian 1 had a greater ability to form higher cellulose content under the coupling of LP and shading (Table 3) [5], it was indicated that the relative cool temperature-tolerant cultivar, such as Kemian 1, still had a greater adaptability to the coupling of LP and shading than the cool temperature-sensitive cultivar, such as Sumian 15.

## Conclusions

(1) The coupling of LP (mainly MDTmin decreased) and shading (*CRLR* 80% and *CRLR* 60%) affected the key enzymes activities (SuSy, SPS, acidic/alkaline INV and CesA) involved in fiber sucrose metabolism and cellulose synthesis and hindered the flux from sucrose to cellulose during FSWD. As for the four sucrose metabolism enzymes (SuSy, SPS, acidic/alkaline INV), effects of LP were greater than shading. The decline of SPS activity and its peak delay probably caused cellulose synthesis being more sensitive to the coupling stress during the later stage of FSWD (38–45 DPA).

(2) LP and shading combined to produce a more severe impact on cellulose synthesis than either stress alone. In the earlier stage of cotton FSWD (before 24 DPA), sucrose contents in cotton fiber under the coupling of LP and shading were mainly used for synthesizing callose instead of synthesizing cellulose. In the later stage of cotton FSWD (after 38 DPA), carbon in fiber stagnated in the form of sucrose and the partitioning to cellulose synthesis decreased, and the coupling of LP and shading made it more serious.

(3) Due to a less sensitive INV, SPS and CesA, the relative cool temperature-tolerant cultivar Kemian 1 had a relatively higher tolerance to the coupling of LP and shading compared to the cool temperature-sensitive cultivar Sumian 15.

## Supporting Information

Figure S1 **Changes of sucrose synthase activities in cotton fiber of two cultivars under the coupling of planting date and shading in 2010 and 2011.** The fiber growing period delineated by the two dotted lines stand for the period of fiber secondary wall rapid-thickening (FSWR).
(TIF)

Figure S2 **Changes of acidic invertase activities in cotton fiber of two cultivars under the coupling of planting date and shading in 2010 and 2011.** The fiber growing period delineated by the two dotted lines stand for the period of fiber secondary wall rapid-thickening (FSWR).
(TIF)

Figure S3 **Changes of alkaline invertase activities in cotton fiber of two cultivars under the coupling of planting date and shading in 2010 and 2011.** The fiber growing period delineated by the two dotted lines stand for the period of fiber secondary wall rapid-thickening (FSWR).
(TIF)

Figure S4 **Changes of sucrose phosphate synthase activities in cotton fiber of two cultivars under the coupling of planting date and shading in 2010 and 2011.** The fiber growing period delineated by the two dotted lines stand for the period of fiber secondary wall rapid-thickening (FSWR).
(TIF)

Table S1 **Variance analysis of mean air temperature, mean relative humidity and photosynthetically active radiation (PAR) in the cotton field under the coupling of planting date and shading in 2010 and 2011.** Data in Table S1 are averaged by measurement data from 6:00am to 6:00pm and PAR was measured at the position about 0.2 m above the canopy. *CRLR* and DPA stand for crop relative light rates and days post anthesis, respectively. Values followed by a different small letter within the same column in the same planting date are significantly different at 0.05 probability level.
(DOC)
