# Supplementary material for: Effect of Late Planting and Shading on Cellulose Synthesis during Cotton Fiber Secondary Wall Development
Source: PLoS One. 2014 Aug 18;9(8):e105088. doi: 10.1371/journal.pone.0105088 (PMC4136859; doi:10.1371/journal.pone.0105088)
Supplement: Table S1 — Variance analysis of mean air temperature, mean relative humidity and photosynthetically active radiation (PAR) in the cotton field under the coupling of planting date and shading in 2010 and 2011. Data in Table S1 are averaged by measurement data from 6:00am to 6:00pm and PAR was measured at the position about 0.2 m above the canopy. CRLR and DPA stand for crop relative light rates and days post anthesis, respectively. Values followed by a different small letter within the same column in the same planting date are significantly different at 0.05 probability level. (DOC) [file pone.0105088.s005.doc]

**Table S1** Variance analysis of mean air temperature, mean relative humidity and photosynthetically active radiation (PAR) in the cotton field under the coupling of planting date and shading in 2010 and 2011.

| Planting dates | *CRLR* (%) | Mean air temperature (^o^C) | | | Mean relative humidity (%) | | | PAR (μmol m^-2^ s^-1^) | | |
| --- | --- | --- | --- | --- | --- | --- | --- | --- | --- | --- |
|  |  | 15 DPA | 30 DPA | 45 DPA | 15 DPA | 30 DPA | 45 DPA | 15 DPA | 30 DPA | 45 DPA |
| **2010** |  |  |  |  |  |  |  |  |  |  |
| 25-Apr | 100 | 34.2 a | 31.6 a | 27.8 a | 64.2 a | 58.5 a | 64.2 a | 734 a | 685 a | 633 a |
|  | 80 | 34.0 a | 31.6 a | 28.4 a | 64.1 a | 62.7 a | 61.3 a | 585 b | 531 b | 491 b |
|  | 60 | 34.0 a | 31.3 a | 28.3 a | 67.7 a | 60.0 a | 60.7 a | 486 c | 419 c | 398 c |
| 25-May | 100 | 30.5 a | 24.2 a | 24.4 a | 62.0 a | 61.4 a | 62.9 a | 639 a | 626 a | 609 a |
|  | 80 | 30.2 a | 24.0 a | 24.5 a | 64.5 a | 60.0 a | 66.2 a | 517 b | 520 b | 484 b |
|  | 60 | 30.3 a | 24.3 a | 24.2 a | 64.5 a | 57.8 a | 66.8 a | 394 c | 369 c | 367 c |
| 10-Jun | 100 | 23.2 a | 23.4 a | - | 58.8 b | 63.7 a | - | 534 a | 578 a | - |
|  | 80 | 23.5 a | 23.0 a | - | 57.1 b | 58.8 a | - | 407 b | 434 b | - |
|  | 60 | 22.6 a | 22.9 a | - | 64.5 a | 59.7 a | - | 311 c | 358 c | - |
| **2011** |  |  |  |  |  |  |  |  |  |  |
| 25-Apr | 100 | 33.4 a | 32.7 a | 32.2 a | 68.1 a | 54.7 b | 69.3 a | 654 a | 764 a | 670 a |
|  | 80 | 33.3 a | 33.0 a | 32.2 a | 66.4 a | 55.5 b | 70.2 a | 501 b | 613 b | 489 b |
|  | 60 | 33.2 a | 32.7 a | 32.5 a | 67.2 a | 59.2 a | 69.7 a | 422 c | 491 c | 393 c |
| 25-May | 100 | 30.9 a | 25.0 a | 26.3 a | 54.7 b | 48.8 a | 61.7 a | 687 a | 589 a | 525 a |
|  | 80 | 31.8 a | 25.5 a | 26.4 a | 55.5 b | 50.7 a | 63.0 a | 535 b | 466 b | 399 b |
|  | 60 | 31.1 a | 25.6 a | 26.4 a | 59.2 a | 52.2 a | 61.1 a | 438 c | 369 c | 331 c |
| 10-Jun | 100 | 25.8 a | 26.5 a | 24.2 a | 46.9 a | 60.2 a | 52.7 a | 568 a | 548 a | 506 a |
|  | 80 | 26.1 a | 25.8 a | 24.2 a | 47.6 a | 60.8 a | 51.8 a | 437 b | 429 b | 389 b |
|  | 60 | 26.2 a | 26.7 a | 24.5 a | 48.7 a | 62.5 a | 52.7 a | 336 c | 346 c | 312 c |

Data in Table S1 are averaged by measurement data from 6:00am to 6:00pm and PAR was measured at the position about 0.2m above the canopy. *CRLR* and DPA stand for crop relative light rates and days post anthesis, respectively. Values followed by a different small letter within the same column in the same planting date are significantly different at 0.05 probability level.
